# Genetic associations between gut microbiota and allergic rhinitis: an LDSC and MR analysis

**DOI:** 10.3389/fmicb.2024.1395340

**Published:** 2024-05-24

**Authors:** XuWen Zheng, MaoBing Chen, Yi Zhuang, Jin Xu, Liang Zhao, YongJun Qian, WenMing Shen

**Affiliations:** Emergency Department, Wujin People’s Hospital Affiliated with Jiangsu University and Wujin Clinical College of Xuzhou Medical University, Changzhou, Jiangsu, China

**Keywords:** gut microbiota, allergic rhinitis, Mendelian randomization, linkage disequilibrium score regression, meta-analysis

## Abstract

**Background:**

Several studies have suggested a potential link between allergic rhinitis (AR) and gut microbiota. In response, we conducted a meta-analysis of Linkage Disequilibrium Score Regression (LDSC) and Mendelian randomization (MR) to detect their genetic associations.

**Methods:**

Summary statistics for 211 gut microbiota taxa were gathered from the MiBioGen study, while data for AR were sourced from the Pan-UKB, the FinnGen, and the Genetic Epidemiology Research on Aging (GERA). The genetic correlation between gut microbiota and AR was assessed using LDSC. The principal estimate of causality was determined using the Inverse-Variance Weighted (IVW) method. To assess the robustness of these findings, sensitivity analyses were conducted employing methods such as the weighted median, MR-Egger, and MR-PRESSO. The summary effect estimates of LDSC, forward MR and reverse MR were combined using meta-analysis for AR from different data resources.

**Results:**

Our study indicated a significant genetic correlation between genus *Sellimonas* (Rg = −0.64, *p* = 3.64 × 10^−5^, Adjust_*P* = 3.64 × 10^−5^) and AR, and a suggestive genetic correlation between seven bacterial taxa and AR. Moreover, the forward MR analysis identified genus *Gordonibacter*, genus *Coprococcus2*, genus *LachnospiraceaeUCG010*, genus *Methanobrevibacter*, and family *Victivallaceae* as being suggestively associated with an increased risk of AR. The reverse MR analysis indicated that AR was suggestively linked to an increased risk for genus *Coprococcus2* and genus *RuminococcaceaeUCG011*.

**Conclusion:**

Our findings indicate a causal relationship between specific gut microbiomes and AR. This enhances our understanding of the gut microbiota’s contribution to the pathophysiology of AR and lays the groundwork for innovative approaches and theoretical models for future prevention and treatment strategies in this patient population.

## 1 Introduction

Allergic rhinitis (AR) affects a significant portion of the global population, with variable prevalence across different regions. Recent surveys in China, for example, report a self-reported AR prevalence of 17.6%, highlighting its substantial impact on public health (Zhang and Akdis, 2022). The symptoms of AR include nasal congestion, watery eyes, rhinorrhea (runny nose), and non-nose/eye symptoms such as itching and sneezing, which significantly impair the quality of life of affected individuals (Li et al., 2022). Beyond the immediate symptoms, AR poses broader implications for human health, contributing to complications such as sleep disturbances, decreased productivity, and an overall reduction in quality of life. The societal impact is also considerable, with substantial healthcare costs and lost productivity (González-Núñez et al., 2013). The pathogenesis of AR is complex, involving both genetic and environmental factors (Wang, 2005). The immune system’s response to allergens plays a crucial role, with IgE-mediated sensitization to common allergens being a central aspect (Eifan and Durham, 2016).

Among the most promising domains in AR research is the involvement of the microbiome as a potential environmental contributor. Viruses and fungi have been reported to be correlated with various allergic conditions. For example, temperate gut phage taxa, particularly the joint abundances of 19 caudoviral families, were associated with later development of asthma (Leal Rodríguez et al., 2024); Proteases and chitin, produced by fungi such as *Alternaria*, *Aspergillus*, and *Cladosporium* were capable of inducing type 2 immune responses via toll-like receptor 4 (TLR4) (Zheng and Dang, 2023). Insights into the gut microbiota’s role in modulating immune responses have opened another avenue for understanding AR’s etiology. Distinct differences in the gut microbiota composition between individuals with AR and healthy controls suggest a potential gut-nose axis, where microbial dysbiosis may influence the development and severity of allergic responses (Su et al., 2021). Moreover, considerable research has been dedicated to exploring the relationship between gut microbiota and AR, investigating how variations in the intestinal flora may predispose to or protect against the disease. Studies employing probiotics, such as *Lactobacillus reuteri* CCFM1040, have demonstrated promising results in alleviating AR symptoms, thereby supporting the hypothesis of a modulatory effect of gut microbiota on allergic diseases (Li et al., 2022). However, establishing causation in these relationships remains challenging due to the potential for confounding factors and reverse causality in observational studies.

In the last few years, advances in genome-wide association studies (GWAS) have led to the development of new statistical approaches for analyzing the relationship and causality among various traits. Among these methods, Linkage Disequilibrium Score Regression (LDSC) is notable for its ability to evaluate genetic correlations using summary statistics from GWAS without being affected by overlapping samples (Bulik-Sullivan B. et al., 2015). Additionally, the use of genetic variations as instrumental variables (IVs) is central to the practice of Mendelian randomization (MR), an epidemiological method aimed at improving the reliability of causal conclusions (Burgess and Thompson, 2015). This approach provides two main advantages: it helps to overcome the issue of confounding variables and reduces the possibility of reverse causation, mainly because genetic variants are allocated randomly at the time of conception. Consequently, these variants are impervious to both self-selected environmental factors and the influences of disease onset or progression (Burgess and Thompson, 2015). A notable MR study, drawing on data from the FinnGen involving individuals of European ancestry (8,430 cases and 298,829 controls), identified a positive causal link between *family Victivallaceae* and AR and a negative causal link between *class Coriobacteriia* and AR (Jin et al., 2023). Despite these advances, significant gaps remain in our understanding of the specific genetic mechanisms that underlie the association between gut microbiota and AR. Many studies have been limited by cross-sectional designs, small sample sizes, or lack of replication across diverse populations. Moreover, there is a need for comprehensive analyses that can provide causal evidence to support observational findings (Stein et al., 2016). Filling these gaps is vital for several reasons. First, it could lead to the identification of biomarkers for early detection and risk stratification of AR. Second, understanding the genetic basis of the gut microbiota’s influence on AR could inform the development of personalized interventions, such as microbiome-based therapies, to prevent or treat AR (West et al., 2015).

Therefore, we undertook a meta-analysis combining LDSC and MR methods to explore the genetic associations between gut microbiota and AR risk, utilizing multiple database sources. The investigation into the genetic underpinnings of AR, particularly in relation to the gut microbiota, holds considerable promise for unveiling new therapeutic targets and prevention strategies. This research is crucial because it bridges a critical knowledge gap in understanding how alterations in gut microbiota composition may influence the development of AR, thereby offering novel insights into disease pathogenesis and potential treatment avenues (Arrieta et al., 2014).

## 2 Materials and methods

### 2.1 Study design

**Figure 1:**
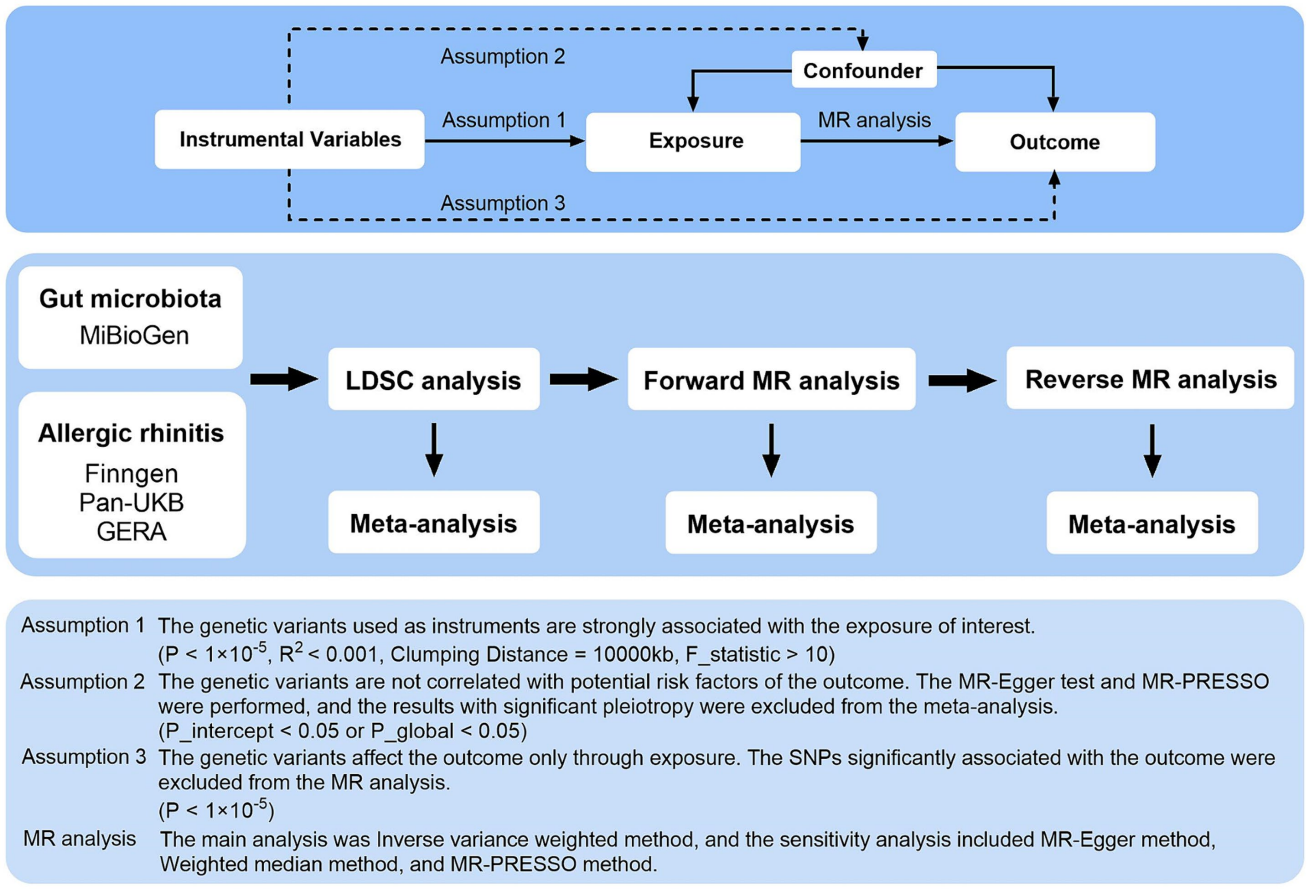
Three assumptions of MR analysis and overview of the study design. MR, Mendelian randomization; GERA, genetic epidemiology research on aging; LDSC, linkage disequilibrium score regression; MR-PRESSO, MR pleiotropy residual sum and outlier; SNPs, single nucleotide polymorphisms.

Figure 1 illustrates the overarching design of this investigation, wherein comprehensive LDSC and MR analyses were conducted to elucidate the relationship between 211 gut microbiota taxa and AR, utilizing three major data sources. This research utilized publicly accessible GWAS, with IVs adhering to three critical assumptions for the execution of MR analysis: (1) The genetic variants serving as instruments must exhibit a strong association with the exposure of interest; (2) These variants should not be associated with any potential risk factors for the outcome; and (3) The influence of the genetic variants on the outcome should be mediated exclusively through the exposure (Burgess and Thompson, 2015). Meta-analysis integrated the summary effect estimates from LDSC, forward MR, and reverse MR to assess AR across various data sources. All included studies received approval from their respective institutional review boards and ethical committees, ensuring that consent forms were obtained from all participants.

### 2.2 Instrumental variable selection

Genetic variations linked to the composition of the gut microbiota were identified through the most extensive genome-wide meta-analysis conducted so far, undertaken by the MiBioGen consortium (Kurilshikov et al., 2021). This analysis encompassed 18,340 participants across 24 cohorts, predominantly of European descent (*n* = 13,266). From the MiBioGen database, 211 gut microbiota taxa were identified, including 12 unknown genera and 3 unknown families. Due to their small portion of the whole, we did not exclude the unknown ones from our analyses. But we would not report the results originating from unknown bacterial taxa. To align with the first assumption of MR analysis, a significance threshold of *p* < 1 × 10^−5^ was employed for gut microbiota identified through GWAS, acknowledging that this rarely meets the genome-wide significance threshold (*p* < 10^−8^) (Sanna et al., 2019). Furthermore, to adhere to MR’s requirement of no linkage disequilibrium (LD) among IVs, IVs were selected based on *R*^2^ < 0.001 and a clumping distance of 10,000 kb to maintain independent single nucleotide polymorphisms (SNPs). To mitigate the influence of weak IVs, the *F*-statistic (*F* = beta^2^/se^2^) was calculated for each gut microbiota taxon, discarding IVs with an *F*-statistic <10 as weak (Bowden et al., 2019; Xie et al., 2023). Harmonization of SNPs in both the exposure and outcome datasets was performed to match alternative and reference alleles, thus eliminating SNPs with mismatched alleles to reduce inconsistencies. Ambiguous palindromic SNPs with minor allele frequencies close to 0.5 were excluded from the MR analyses. For the second assumption, MR-Egger intercept test and MR pleiotropy residual sum and outlier (MR-PRESSO) test were conducted to identify pleiotropy, excluding MR estimates with significant pleiotropy from the meta-analysis (*P* for intercept <0.05 or *P* for global test <0.05). Lastly, for the third assumption, SNPs significantly associated with the outcome (*p* < 1 × 10^−5^) were omitted from the MR analysis to ensure the validity of causal inferences. The instrumental variables associated with all 211 gut microbiota taxa were comprehensively listed in Supplementary Table S1.

### 2.3 AR data sources

Summary-level data for AR were derived from three major sources: the Pan-UKB GWAS Version 0.4, released on March 16, 2023 (Pan-UKB Team, 2020); the FinnGen GWAS Release 10, released on December 18, 2023 (Kurki et al., 2023); and the Genetic Epidemiology Research on Aging (GERA) (Guindo-Martínez et al., 2021). The total sample size encompassed 27,707 cases and 833,527 controls of European ancestry. The Pan-UKB GWAS utilized data from the UK Biobank, an extensive open-access database containing genotype information for hundreds of thousands of individuals, alongside with electronic health records and survey responses, aimed at studying populations of diverse ancestries (Pan-UKB Team, 2020). For this analysis, we specifically employed summary statistics for European ancestry from the Pan-UKB team, where AR was categorized under 476 according to the phecode. The FinnGen GWAS represents a comprehensive national genetic study, integrating genetic data with electronic health records (Kurki et al., 2023). AR was identified according to the International Classification of Diseases, 10th Revision (ICD-10) (J30.1, J30.19, J30.2, J30.3, and J30.4) and ICD-9 (477) classifications. For the GERA data, diagnosis of AR cases adhered to ICD-9 (477) (Guindo-Martínez et al., 2021). Detailed descriptions of the studies utilized are provided in Table 1.

**Table 1:** Detailed information on used summary-level data.

Exposure or outcome	Consortium	Participants included in analysis	Age and sex	Adjustments	Web source
Gut microbiota	MiBioGen	18,340 multiple-descent individuals		Age, sex, technical covariates and genetic principal components	https://mibiogen.gcc.rug.nl/
Allergic rhinitis	FinnGen	12,240 cases and 392,069 controls of European ancestry	Median age of 63 years for both males and females	Sex, age, genotyping batch and 10 principal components	https://r10.finngen.fi/
	Pan-UKB	1,531 cases and 398,757 controls of European ancestry	Average age of 56 years for both males and females	Age, sex, age*sex, age^2^, age^2^*sex, first 10 genetic principal components	https://pan.ukbb.broadinstitute.org/downloads/
	GERA	13,936 cases and 42,701 controls of European ancestry	Average age of 63 years for both males and females	Seven derived principal components, sex, and age	http://cg.bsc.es/gera_summary_stats/

### 2.4 Statistical analysis

We conducted an analysis to determine the genetic correlation between gut microbiota and AR using LDSC. The GWAS summary data were refined using HapMap3 references, with the exclusion of non-SNP variants such as insertions and deletions (indels) and SNPs that were ambiguous in terms of strand orientation, duplicated, or exhibited a minor allele frequency lower than 0.01. LDSC is capable of quantifying genetic correlation using GWAS summary statistics. It assesses the relationship between LD and test statistics to identify whether observed inflation is due to genuine polygenic signals or other biases (Bulik-Sullivan B. K. et al., 2015). This approach is unaffected by sample overlap (Bulik-Sullivan B. et al., 2015). Genetic covariance is calculated by multiplying the *z*-scores of variants associated with Trait 1 by those associated with Trait 2, and subsequently regressing these products against the LD score (Wielscher et al., 2021). After adjusting this covariance by SNP heritability, the genetic correlation becomes clear. Estimates of genetic correlation between gut microbiota and AR from three data sources were combined through fixed-effects meta-analysis.

For causal analysis, the major MR estimate was computed using the inverse-variance weighted (IVW) method under a random-effects model. To assess the presence of horizontal pleiotropy and validate the data’s reliability, we conducted three sensitivity analyses: weighted median, MR-Egger, and MR-PRESSO. SNP heterogeneity was assessed via the Cochran *Q* value. The MR-Egger intercept test identifies horizontal pleiotropic effects, and after adjusting for such effects, the MR-Egger method offers estimates, albeit with reduced precision (Burgess and Thompson, 2017). MR-PRESSO identifies and adjusts for horizontal pleiotropic outliers, allowing for refined estimations post-outlier removal. To detect horizontal pleiotropy, the global test was applied, with the distortion test comparing pre-and post-outlier removal estimates (Verbanck et al., 2018). Combined estimates from IVW and sensitivity analyses were integrated using fixed-effects meta-analysis. Exposures represented by fewer than four SNPs were omitted from the analysis, as MR-PRESSO requires a minimum of four instrumental SNPs. Estimates indicating significant pleiotropy (*P* for intercept test <0.05 or *P* for global test <0.05) were also excluded from the meta-analysis.

The Benjamini–Hochberg correction, a procedure designed to minimize the false discovery rate, was applied to adjust for multiple comparisons in meta-analyses conducted for both forward and reverse MR analyses separately. An MR association with an IVW *p*-value less than 0.05 and consistent with all sensitivity analyses was deemed suggestive. The suggestive association was deemed significant under one of the following criteria: (1) Benjamini–Hochberg adjusted *p*-value less than 0.05 (Benjamini and Hochberg, 1995); or (2) LDSC correlation with a *p*-value less than 0.05, provided both outcomes were consistent. All statistical analyses were performed using R software (version 4.3.1), utilizing the TwoSampleMR, GenomicSEM, and meta packages.

## 3 Results

### 3.1 LDSC analysis

**Figure 2:**
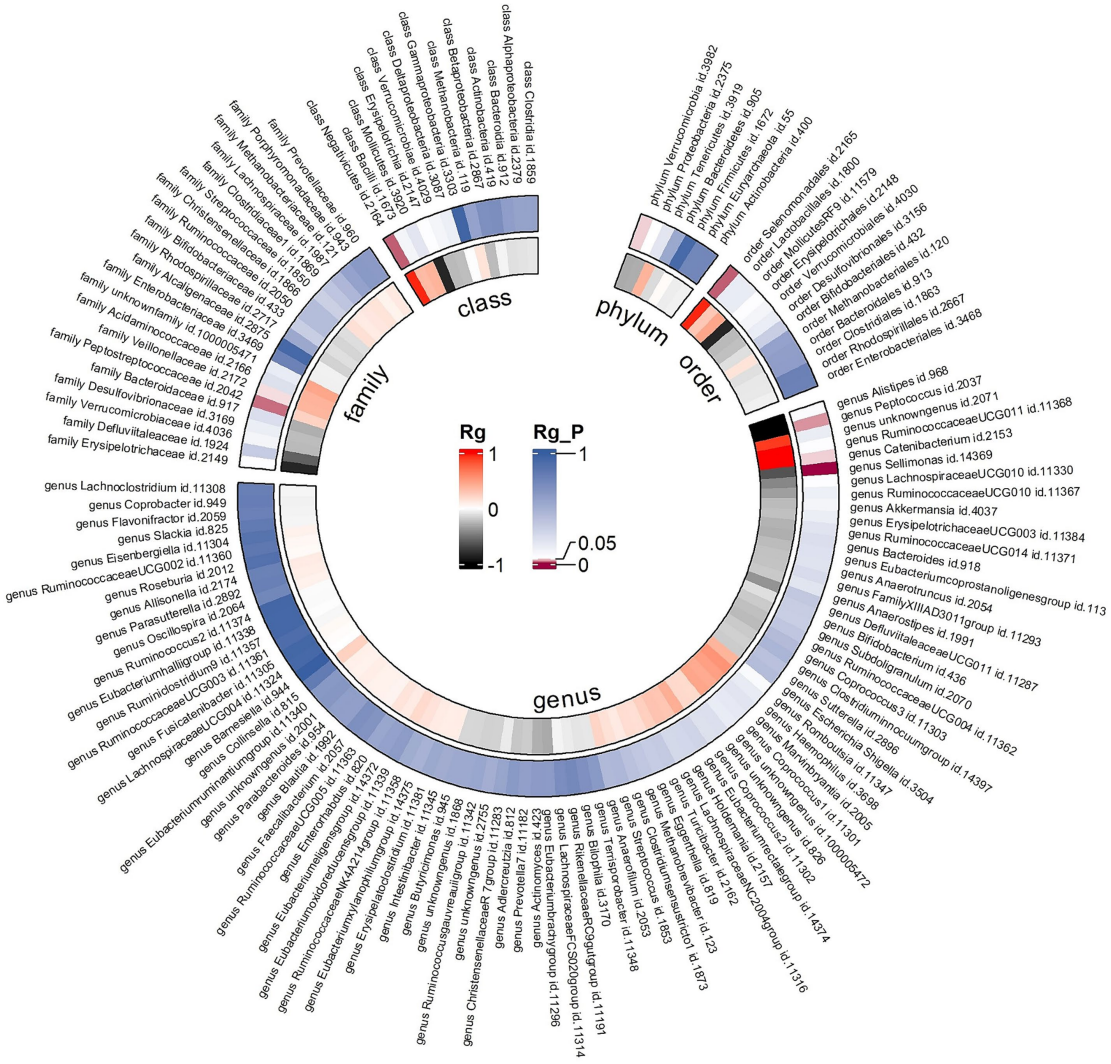
Circular heat map of meta-analysis of genetic correlation between gut microbiota and allergic rhinitis. Rg, estimate of genetic correlation; Rg_*P*, *p*-value for Rg.

Due to constraints like low heritability and small sample sizes, certain bacterial taxa are not suitable for the analysis mentioned above. We performed a meta-analysis of LDSC to evaluate the genetic correlation between 137 gut microbes and AR, including 7 unknown taxa (Figure 2). As shown in Table 2, LDSC showed a significant negative correlation between genetically predicted genus *Sellimonas* and AR (Rg = −0.64, *p* = 3.64 × 10^−5^, Adjust_*P* = 3.64 × 10^−5^). Moreover, genetic predisposition to class *Negativicutes* (Rg = 0.96, *p* = 0.014), order *Selenomonadales* (Rg = 0.06, *p* = 0.014), family *Peptostreptococcaceae* (Rg = 0.23, *p* = 0.018), genus *Catenibacterium* (Rg = 1.10, *p* = 0.04), and family *Veillonellaceae* (Rg = 0.38, *p* = 0.043) were suggestively positively correlated with AR, and genetic predisposition to genus *Peptococcus* (Rg = −1.21, *p* = 0.026) and phylum *Verrucomicrobia* (Rg = −0.30, *p* = 0.04) were suggestively negatively correlated with AR. No heterogeneity or mild heterogeneity was observed across most of the results. Detailed information regarding all genetic correlation results is listed in Supplementary Table S2.

**Table 2:** Meta-analysis of genetic correlation between gut microbiota and allergic rhinitis from three large databases.

Exposure	Rg	Rg_Se	Rg_*P*	Adjust_*P*	*I* ^2^	*P*_heterogeneity
Genus *Sellimonas*	−0.644	0.156	3.64E-05	3.64E-05	0.338	0.221
Class *Negativicutes*	0.958	0.391	0.014	1.000	0	0.551
Order *Selenomonadales*	0.958	0.391	0.014	0.984	0	0.551
Family *Peptostreptococcaceae*	0.228	0.096	0.018	0.812	0.530	0.119
Genus *Peptococcus*	−1.214	0.546	0.026	0.899	0	0.523
Phylum *Verrucomicrobia*	−0.302	0.147	0.040	1.000	0.298	0.241
Genus *Catenibacterium*	1.099	0.535	0.040	0.907	0	0.513
Family *Veillonellaceae*	0.380	0.187	0.043	0.834	0	0.681

### 3.2 Forward MR analysis

After the instrumental variable selection procedure, one bacterial genus (genus *LachnospiraceaeND3007group*) was excluded from the MR analysis due to less than four SNPs. Then, meta-analyses of 210 gut bacteria were conducted, including 15 unknown taxa (Supplementary Table S3). Finally, we identified five bacterial taxa suggestively associated with AR.

**Figure 3:**
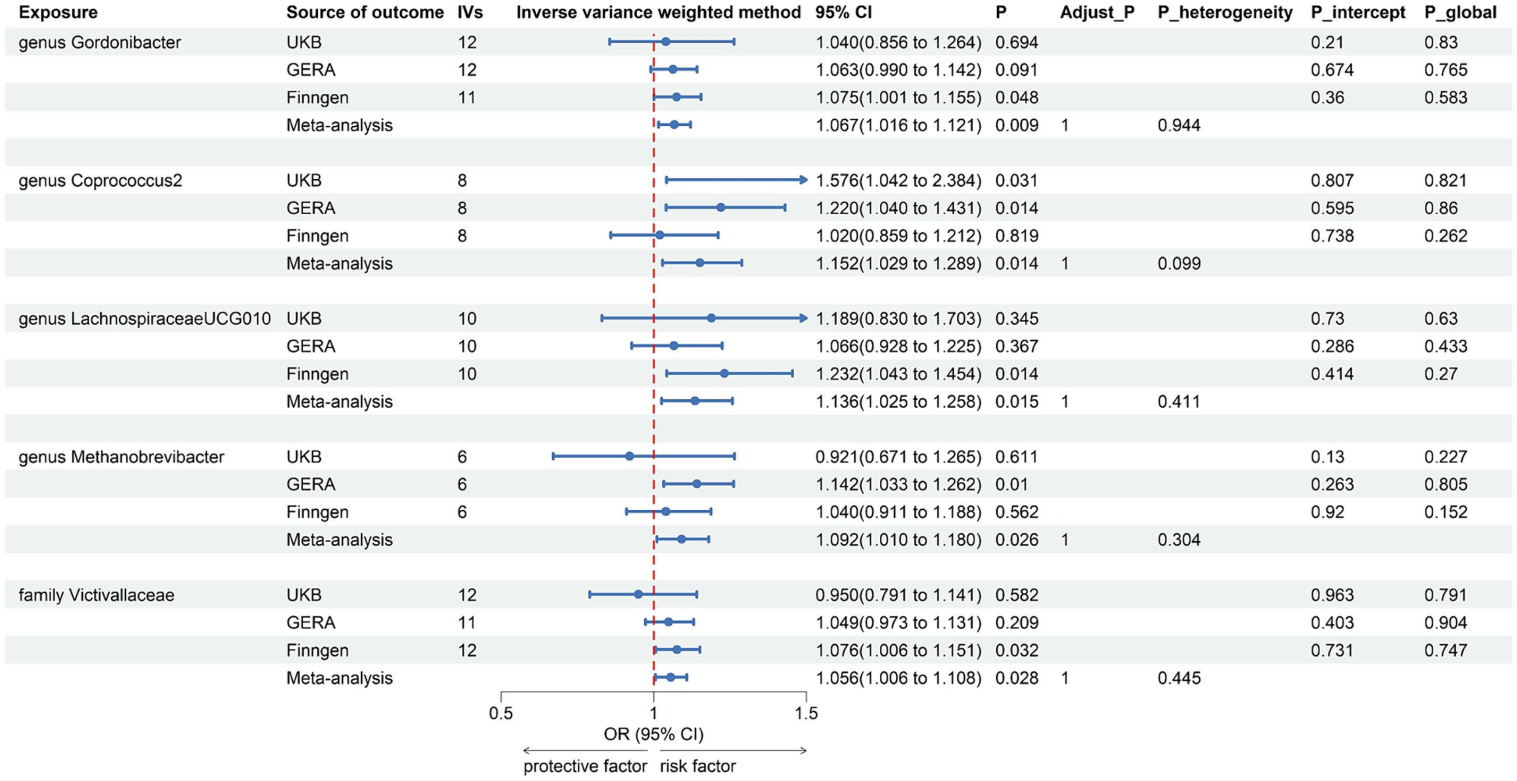
Forest plot of suggestive associations in forward MR analysis. IVs, instrumental variables; CI, confident interval; Adjust_*P*, *p*-value after Benjamini–Hochberg correction; *P*_heterogeneity, *p*-value for heterogeneity in meta-analysis; *P*_intercept, *p*-value for MR-Egger intercept test; *P*_global, *p*-value for Global test.

**Figure 4:**
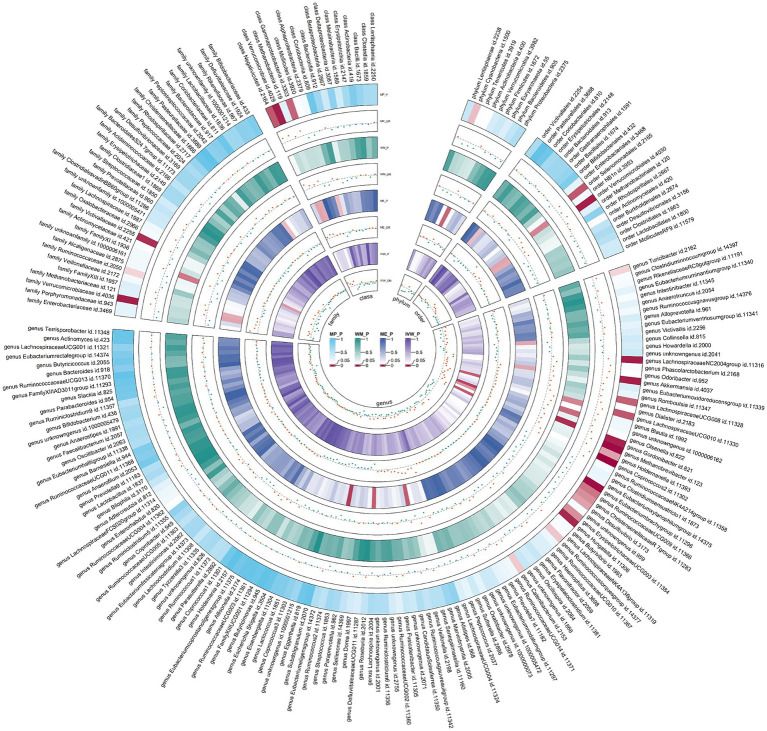
Circular heat map of meta-analysis of forward MR analysis between gut microbiota and allergic rhinitis. IVW, inverse-variance weighted; ME, MR-Egger; WM, weighted median; MP, MR-PRESSO. The color variations represented the size of the *p*-value. The scatter plots reflect OR, with OR > 1 labeled red and OR < 1 labeled green.

The combined results of IVW method revealed that genetic predisposition to genus *Gordonibacter* (OR = 1.067, 95% CI 1.016, 1.121; *p* = 0.009), genus *Coprococcus2* (OR = 1.152, 95% CI 1.029, 1.289; *p* = 0.014), genus *LachnospiraceaeUCG010* (OR = 1.136, 95% CI 1.025, 1.258; *p* = 0.015), genus *Methanobrevibacter* (OR = 1.092, 95% CI 1.010, 1.180; *p* = 0.026), and family *Victivallaceae* (OR = 1.056, 95% CI 1.006, 1.108; *p* = 0.028) were suggestively associated with an increased risk of AR (Figure 3). The abovementioned associations were consistent with all sensitivity analyses. The Cochran *Q* test, which was used to evaluate SNP estimates of heterogeneity, did not detect any heterogeneity in the MR estimates combined within the abovementioned meta-analysis estimates. Pleiotropy did not need to be considered in this study due to the removal of the estimates with significant pleiotropy. No heterogeneity or mild heterogeneity was observed across most of the results. All the combined estimates are depicted in Figure 4.

### 3.3 Reverse MR analysis

**Figure 5:**
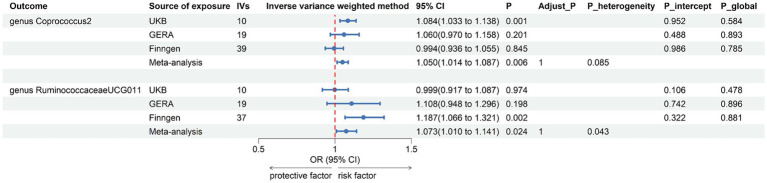
Forest plot of suggestive associations in reverse MR analysis. IVs, instrumental variables; CI, confident interval; Adjust_*P*, *p*-value after Benjamini–Hochberg correction; *P*_heterogeneity, *p*-value for heterogeneity in meta-analysis; *P*_intercept, *p*-value for MR-Egger intercept test; *P*_global, *p*-value for Global test.

Following the same IVs selection procedure for gut microbiota, all 211 meta-analyses were conducted, and the results indicated that AR was suggestively associated with two bacterial taxa (Figure 5 and Supplementary Table S4).

**Figure 6:**
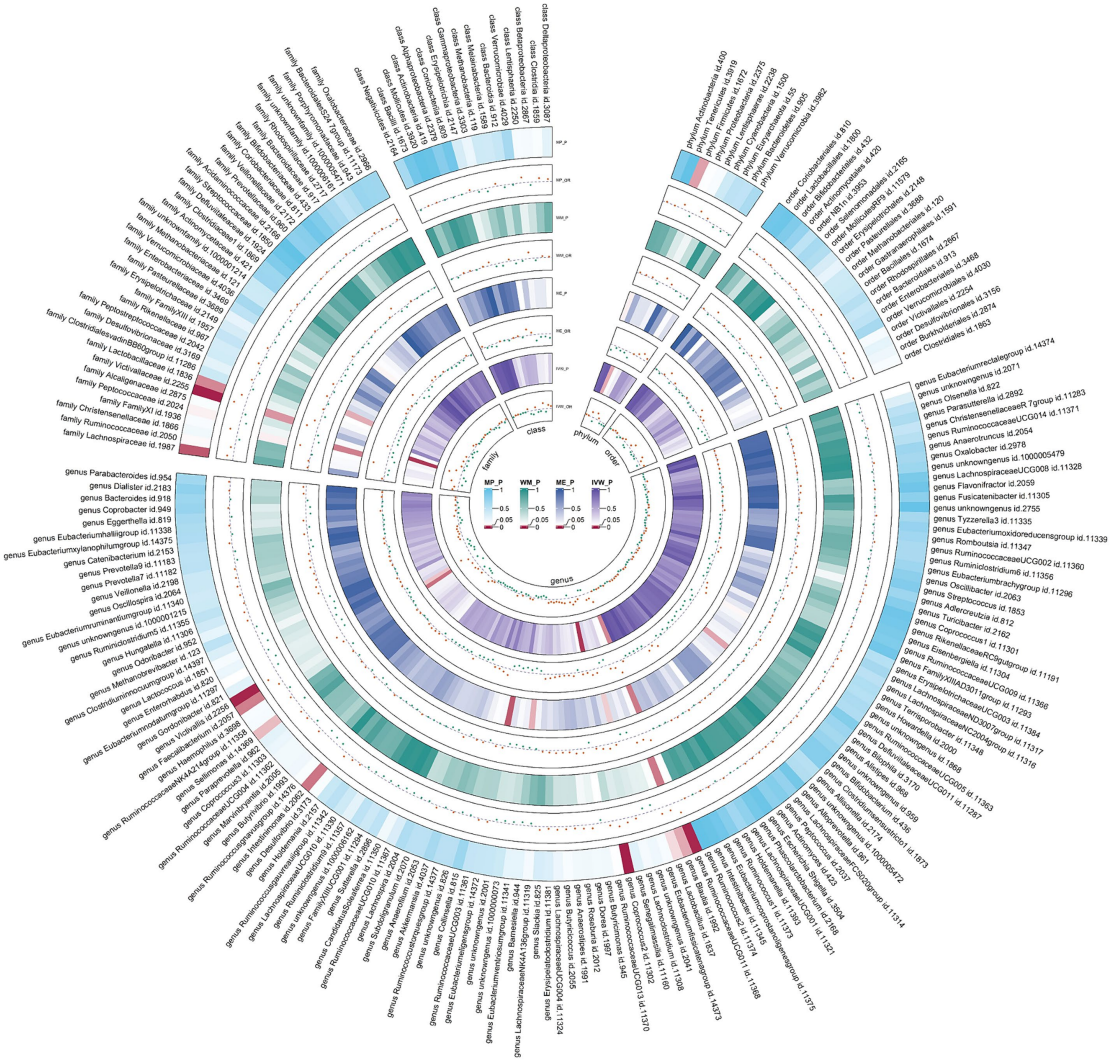
Circular heat map of meta-analysis of reverse MR analysis between gut microbiota and allergic rhinitis. IVW, inverse-variance weighted; ME, MR-Egger; WM, weighted median; MP, MR-PRESSO. The color variations represented the size of the *p*-value. The scatter plots reflect OR, with OR > 1 labeled red and OR < 1 labeled green.

The combined results of IVW method revealed that genetically predicted AR was suggestively associated with an increased risk of genus *Coprococcus2*, family *Victivallaceae*, genus *Faecalibacterium*, genus *RuminococcaceaeUCG011*, phylum *Firmicutes*, and genus *Victivallis*. However, only in genus *Coprococcus2* (OR = 1.050, 95% CI 1.014, 1.087; *p* = 0.006) and genus *RuminococcaceaeUCG011* (OR = 1.073, 95% CI 1.010, 1.141; *p* = 0.024) were the combined results consistent with all sensitivity analyses. The Cochran *Q* test did not detect any heterogeneity in the MR estimates combined within the abovementioned meta-analysis estimates. Pleiotropy did not need to be considered due to our study design. No heterogeneity or mild heterogeneity was observed across most of the results. All the combined estimates are depicted in Figure 6. The bilateral MR analysis identified a suggestive bidirectional causality between genus *Coprococcus2* and AR.

## 4 Discussion

Our investigation delved into the genetic link and possible causative relationships between gut microbiota and AR through the analysis of GWAS summary statistics. The analysis revealed a significant correlation between genus *Sellimonas* and AR and a suggestive genetic correlation between seven bacterial taxa and AR. Additionally, the MR analysis suggested causal associations between five bacterial taxa and AR, as well as AR being suggestively causally linked to two bacterial taxa. These insights pave the way for potential microbiological interventions in upcoming clinical trials targeting AR.

Within the gut microbiota community, the phyla *Firmicutes*, *Bacteroidetes*, *Proteobacteria*, and *Actinobacteria* collectively account for over 90% of its composition (Bibbò et al., 2016). The multifunctional roles of the gut microbiota have led to persuasive evidence that its dysregulation may be intricately linked to low-grade inflammation and a spectrum of pathologies, including allergic conditions like AR (Ipci et al., 2017). For example, research by Zhu et al. (2020) reported that individuals with AR exhibit distinct gut microbiota characteristics compared to healthy controls, suggesting the gut microbiota’s crucial role in influencing the course and symptoms of AR. Furthermore, research has suggested that dysregulation of the gut microbiota contributes to chronic inflammatory diseases, including allergic asthma and AR, by dysregulating inflammation and affecting the interplay between gut microbiota and host cells (Kim et al., 2017). On the other hand, animal research showed that vancomycin-induced gut microbiota dysbiosis aggravates AR in mice, highlighting the role of short-chain fatty acids (SCFAs) in mediating the effects of gut microbiota dysbiosis on allergic diseases. Supplementation with sodium butyrate, an SCFA, has been shown to alleviate symptoms and improve immune status in AR models (Chen Z. et al., 2022). A reduction in Firmicutes coupled with an increase in Bacteroidetes results in diminished production of SCFAs. This may enhance intestinal permeability, thereby triggering an inflammatory response and potentially elevating the risk of allergic conditions (Yamaguchi et al., 2023; Zhang P. et al., 2023).

The interaction between the microbiome and the immune system is a well-established area of research. Dysbiosis, or the imbalance in the microbial community, is hypothesized to cause abnormal allergic reactions by shifting the immune system toward a Th2 response and IgE production in AR (Li et al., 2023). A landmark paper by Haahtela et al. (2013) discusses how changes in lifestyle and environment over the past century have led to a decrease in microbial diversity and an increase in allergic diseases. Phylum *Verrucomicrobia* includes species that have been associated with health benefits, including anti-inflammatory properties, which could theoretically contribute to their negative correlation with AR (Fujio-Vejar et al., 2017). Additionally, *Sellimonas intestinalis*, a member of genus *Sellimonas*, has been noted for its role in the gut microbiome, particularly in relation to intestinal homeostasis recovery after dysbiosis events. The genome analysis of *S. intestinalis* reveals genes involved in amino acid and carbohydrate transport as well as energy production and conversion, which aligns with the metabolic profile typically associated with a healthy microbiota (Muñoz et al., 2020). A gut homeostasis may influence the maturation and function of immune cells, such as T regulatory cells and Th2 cells, and skew immune responses away from Th2-dominant allergic reactions, thus potentially mitigating the symptoms of AR. The genus *Peptococcus*, now reclassified into different genera such as *Peptoniphilus*, *Anaerococcus*, and *Gallicola*, includes species that have been identified as butyrate-producing (Ezaki et al., 2001). The SCFAs, including acetate, propionate, and butyrate, have been demonstrated to influence the immune system in several ways. They can enhance the production of regulatory T cells (Tregs) and reduce pro-inflammatory cytokines, thus promoting an anti-inflammatory state conducive to protecting against allergic diseases such as AR (Budden et al., 2017). Additionally, SCFAs contribute to maintaining the integrity of the gut barrier, which prevents the translocation of allergens and pathogens that could trigger immune responses leading to allergic diseases. A healthy gut barrier supported by SCFAs may thus indirectly contribute to a reduced risk of AR by limiting systemic exposure to potential allergens (Maslowski and Mackay, 2011). Furthermore, SCFAs, particularly butyrate, have been shown to possess anti-inflammatory properties that can influence the respiratory tract. By reducing inflammation, SCFAs can potentially lower the risk of AR, which is characterized by an inflammatory response to allergens in the nasal passages (Trompette et al., 2014).

The gastrointestinal tract (GIT) and respiratory tract (including oral and nasopharyngeal cavity), although separate organs, are part of a shared mucosal immune system termed the gut-lung axis (GLA). Airway colonization with pathogenic bacteria in early life is associated with an increased risk of respiratory allergic conditions (Bisgaard et al., 2023). Observational studies have identified notable differences in bacterial diversity between AR patients and healthy controls in oral and nasopharyngeal cavity. Specifically, the bacterial phyla *Fusobacteriota* and *Proteobacteria*, along with genera such as *Fusobacterium*, *Gemella*, *Haemophilus*, *Leptotrichia*, *Neisseria*, and *Porphyromonas*, were found to be significantly more prevalent in the oral bacteriomes of AR patients. Conversely, higher concentrations of the phylum *Firmicutes* and the genera *Faecalibacterium*, *Lactobacillus*, and *Escherichia* were observed in the nasal cavities of those with AR (Chen M. et al., 2022; Pérez-Losada et al., 2023). These discrepancies may be influenced not only by diet, environmental pollutants, health status, and genetic factors, but also by microbial interactions (Blais and Lavoie, 1990; Gomez et al., 2017; Samaranayake and Matsubara, 2017; Salzano et al., 2018; Wade, 2021; Yan et al., 2022). The GLA involves host-microbe and microbe-microbe interactions that can shape immune responses and influence the course of respiratory diseases. This crosstalk includes both localized effects within the gastrointestinal and respiratory tract, and long-reaching effects that can affect one organ system based on changes in the other (Enaud et al., 2020). For example, Alterations in the gut microbiota might reflect or affect changes in the oropharyngeal microbiota, which can directly influence lung microbiota and host immune responses, possibly through mechanisms like microaspiration. Furthermore, the gut microbiota, through its diverse microbial composition, can regulate the balance between different types of T cells in the intestines, which can influence systemic immune responses and possibly exacerbate or mitigate allergic responses indirectly (Ipci et al., 2017). However, determining which microbial species influence the oral and nasal microbiomes through these mechanisms remains challenging.

An increased risk of asthma has been associated with the relative abundances of *Lachnospira* spp. and *Veillonella* spp. (Arrieta et al., 2015). As another allergic airway condition, AR was found to be positively associated with family *Veillonellaceae* in our study. This finding may be explained by a pro-inflammatory phenotype characterized by elevated Th-17 lymphocytes and, conversely, a blunted alveolar macrophage TLR4 response (Segal et al., 2016). In an observational study, the average relative abundance of genus *Lachnospira* and genus *LachnospiraceaeUCG008* was significantly different in the AR group compared to the non-AR group, and genus *LachnospiraceaeUCG001* was positively associated with most clinical symptoms of AR (Zhu et al., 2020). In our research, family *Victivallaceae* and genus *LachnospiraceaeUCG010* were identified as risk factors for AR. However, the pathogenic mechanisms between these bacterial taxa and AR are still poorly understood (Jin et al., 2023).

As mentioned above, SCFAs could reduce the occurrence and progression of allergic conditions in several aspects. However, Zhang C. et al. (2023) observed that genus *Gordonibacter* might negatively affect SCFAs, hinting at genus *Gordonibacter* as a risk factor for AR. Moreover, *Gordonibacter pamelaeae*, a species within the genus *Gordonibacter*, was first isolated from the colon of a patient with Crohn’s disease, suggesting its potential pro-inflammatory effects (Würdemann et al., 2009). Genus *Methanobrevibacter*, as a methane-producing microbiome, along with the majority of its species, has been conclusively linked to intestinal motility disorders, such as constipation (Miller and Lin, 2002; Pimentel et al., 2003; Kunkel et al., 2011), which could affect the gut microbiota’s composition and, by extension, systemic immune responses. This altered transit time could influence the development of allergic conditions by impacting the exposure of the gut immune system to microbial antigens and metabolites (Triantafyllou et al., 2014). Our study also identified a suggestive bidirectional causality between genus *Coprococcus2* and AR. The genus *Coprococcus* is known for its butyrate-producing capabilities. Butyrate has anti-inflammatory properties and can modulate immune responses, potentially affecting the development and severity of allergic reactions in the nasal mucosa (Arrieta et al., 2015). Therefore, a bidirectional relationship between *Coprococcus2* and AR suggests that not only can changes in gut microbiota influence AR, but AR or its treatment might also alter gut microbiota composition, further influencing systemic immunity.

Changes in microbial composition and function in the respiratory tract and the gut have recently been linked to alterations in immune responses and to other diseases development in the lungs, such as asthma, chronic obstructive pulmonary disease, and respiratory infections (Rutten et al., 2014; Arrieta et al., 2015; Schuijt et al., 2016). For example, Elevated levels of *Lachnospiraceae* were observed in a smoking-based mouse model of chronic obstructive pulmonary disease (Lai et al., 2022). The relative abundance of *Coprococcus* in the asthma mice was significantly elevated (Gong et al., 2021). Although direct evidence is lacking regarding the impact of the flora we identified on human respiratory diseases, it is reasonable to infer that their effects are not confined to AR but extend to the entire respiratory system.

In a previous MR analysis, families *Bifidobacteriaceae* and *Clostridiaceae*, and genera *Bifidobacterium* and *Anaerostipes* were found to be associated with an increased risk of AR. However, due to limited number of SNPs and sample size, the causal associations could not be replicated in two similar outcome studies (Wang et al., 2023). Our study possesses several strengths. Firstly, its primary advantage lies in the MR design, which significantly mitigates confounding and reverse causality (Burgess and Thompson, 2015). Secondly, by focusing exclusively on individuals of European ancestry, we minimized the risk of population structure bias, although this approach may limit the generalizability of our findings to other populations. The demographic characteristics (age distribution and gender composition) of AR from different databases were comparable; therefore, we believe that these factors had little impact on our results. Thirdly, we employed meta-analysis to enhance the stability of our results, mitigating the variability inherent in relying on a single database. Fourthly, MR estimates affected by significant pleiotropy were omitted from our meta-analysis, thereby increasing the reliability of our results. Finally, we applied the Benjamini-Hochberg correction to reduce the false positive rate associated with multiple analyses, in conjunction with the LDSC correlation *p*-value approach to minimize the false negative rate resulting from multiple adjustments.

Evaluating our study’s findings necessitates acknowledging its limitations. Firstly, the GWSA data concerning the gut microbiota were gathered from a varied group of 18,340 participants spanning multiple ethnic backgrounds, in contrast to the GWAS summary statistics for AR, which came solely from individuals of European ancestry. Nevertheless, given that almost 80% of the data on the gut microbiota from GWAS is sourced from European populations, and considering the dataset’s extensive, diverse, and representative characteristics, it was considered appropriate for our study. Additionally, its extensive use in prior MR studies further validates our choice. Secondly, there are slight discrepancies between different datasets. However the overall heterogeneity remains minimal, which confirms the stability and reliability of our results. Thirdly, the lack of detailed data precluded stratified analyses by age and gender, inhibiting our ability to explore potential differences across various demographics. Fourthly, while our rigorous study design identified certain causal relationships, the complex nature of gut microbiota and its pathogenesis remains partially understood, highlighting a gap for future research to bridge.

## 5 Conclusion

Our results demonstrate a causal link between particular gut microbiomes and AR, enriching our insight into how gut microbiota influence AR’s pathophysiology. The likely involvement of gut microbiota in AR’s pathogenesis heralds innovative strategies for modulating the GLA, including dietary modifications and the application of prebiotics, probiotics, postbiotics, fecal microbiota transplantation, and other lifestyle interventions. Overall, the gut microbiota not only adds a critical dimension to the study of AR but also opens avenues for promising translational research, improved monitoring, and enhanced clinical management and quality of life for affected individuals.

## Data availability statement

The datasets presented in this study can be found in online repositories. The names of the repository/repositories and accession number(s) can be found in the article/Supplementary material. If more information is needed, the corresponding author can be contacted.

## Ethics statement

The studies involving humans were approved by the Coordinating Ethics Committee of the Helsinki and Uusimaa Hospital District has approved the FinnGen GWAS project. The North West-Haydock Ethics Committee, National Health Service (NHS) National Research Ethics Service has approved the Pan-UKB GWAS project. Kaiser Permanente Northern California Division of Research has approved the GERA GWAS project. Twenty-four cohorts in MiBioGen GWAS were all approved by their respective institutional review boards and ethical committees. The studies were conducted in accordance with the local legislation and institutional requirements. Written informed consent for participation was not required from the participants or the participants’ legal guardians/next of kin in accordance with the national legislation and institutional requirements.

## Author contributions

XZ: Methodology, Software, Visualization, Writing – original draft. MC: Formal analysis, Investigation, Visualization, Writing – review & editing. YZ: Investigation, Visualization, Writing – review & editing. JX: Formal analysis, Visualization, Writing – review & editing. LZ: Formal analysis, Investigation, Writing – review & editing. YQ: Formal analysis, Investigation, Visualization, Writing – review & editing. WS: Funding acquisition, Project administration, Resources, Writing – review & editing.
